# Balancing Nutritional Value and Food Safety in Peanut Butter: The Role of Food Matrix Characteristics in Hazard Behavior and Risk Management

**DOI:** 10.3390/foods15162827

**Published:** 2026-08-13

**Authors:** Wojciech Gaworowski, Jakub Ostrzycki, Beata Sperkowska, Marcin Gackowski, Katarzyna Mądra-Gackowska

**Affiliations:** 1 Department of Toxicology and Bromatology, Faculty of Pharmacy, Ludwik Rydygier Collegium Medicum in Bydgoszcz, Nicolaus Copernicus University in Toruń, A. Jurasza 2 Street, 85-089 Bydgoszcz, Poland; w.gaworowski@gmail.com (W.G.); k.ostrzycki@rostry.com.pl (J.O.); marcin.gackowski@cm.umk.pl (M.G.); 2 Department of Geriatrics, Faculty of Health Science, Ludwik Rydygier Collegium Medicum in Bydgoszcz, Nicolaus Copernicus University in Toruń, Skłodowska-Curie 9 Street, 85-094 Bydgoszcz, Poland; katarzyna.madra@cm.umk.pl

**Keywords:** food matrix, low-moisture foods, matrix-dependent hazard behavior, *Salmonella*, aflatoxins, nutritional quality, risk–benefit assessment, food safety

## Abstract

Peanut butter combines nutritional density with two hazard profiles that are often discussed separately: persistence of enteric pathogens in a low-moisture, lipid-rich matrix and pre-processing contamination with aflatoxins. This structured narrative review synthesizes evidence identified in PubMed, Scopus, Web of Science, and Google Scholar between January and June 2026 and asks whether food-matrix characteristics change not only nutrient release but also the reliability and timing of safety controls. The evidence is strongest for prolonged survival and matrix-enhanced heat resistance of *Salmonella*, the heterogeneous distribution of aflatoxins among kernels, and the limited corrective value of roasting for contaminated lots. Direct studies that measure nutritional and safety outcomes within the same peanut butter formulation are scarce; the proposed framework is therefore an integrative interpretation rather than a quantitatively validated model. In our assessment, the practical value of the matrix concept lies in three decisions: controlling aflatoxins before grinding, validating microbial lethality in the actual product, and protecting post-lethality areas from recontamination. These conclusions support hazard-specific risk management that preserves nutritional and sensory quality without treating shelf stability as evidence of microbiological safety.

## 1. Introduction

Peanut butter is one of the most widely consumed peanut-derived products worldwide and has become an established component of contemporary dietary patterns. Its popularity is largely attributed to its favorable nutritional profile, which includes plant protein, predominantly unsaturated fatty acids, dietary fiber, vitamins, minerals, and numerous bioactive compounds [1,2]. Owing to these characteristics, peanut butter is increasingly recognized as a nutrient-dense food and is frequently incorporated into dietary patterns emphasizing plant-based and health-oriented food choices [3,4].

At the same time, peanut butter presents unique food safety challenges that distinguish it from many other foods. *Salmonella* remains one of the most significant foodborne zoonotic pathogens worldwide, transmitted through diverse animal- and plant-derived food vehicles across the farm-to-fork continuum [5]. Despite being classified as a low-moisture product with limited capacity to support microbial growth, peanut butter has repeatedly been implicated in outbreaks of *Salmonella-associated foodborne illness* [6,7]. In addition, peanuts are susceptible to contamination by aflatoxin-producing fungi, creating a significant mycotoxicological concern that may originate long before processing begins [8,9,10]. These observations illustrate the complexity of ensuring both the nutritional value and safety of peanut-derived products.

Traditionally, nutritional quality and food safety have been investigated as largely independent areas of food science. Nutritional research has primarily focused on the health-promoting properties of foods and dietary patterns. In contrast, food safety studies have concentrated on the occurrence, behavior, and control of biological and chemical hazards. However, increasing evidence suggests that these perspectives are more closely interconnected than previously recognized [11,12]. In particular, growing attention has been paid to the food matrix concept, which emphasizes that nutrients, microorganisms, contaminants, and processing effects should be understood in the context of the complex structural organization of foods rather than as isolated components [4,11].

The food matrix concept has emerged as an important framework for explaining why foods with similar nutrient compositions may differ in physiological effects, bioavailability, and nutritional functionality [12,13]—a relationship well illustrated by studies showing that food form, texture, and matrix organization directly modulate energy intake and metabolic responses independent of nutrient composition [14]. Nevertheless, its implications for food safety have received comparatively less attention. Characteristics such as water activity, lipid content, physical structure, and matrix organization may influence not only nutritional outcomes but also microbial survival, thermal resistance, contaminant behavior, and the effectiveness of food safety interventions [6,15]. Consequently, the food matrix may represent a common factor linking nutritional functionality and hazard behavior.

Peanut butter provides a particularly valuable model for exploring these interactions. The product combines substantial nutritional benefits with distinctive microbiological and mycotoxicological challenges, many of which are directly influenced by matrix characteristics [6,7]. As a result, peanut butter offers an opportunity to examine how the same physicochemical properties may simultaneously contribute to both nutritional value and food safety concerns.

Existing literature on peanuts and peanut butter has largely developed along separate lines. Reviews of nutritional chemistry and health effects have focused on nutrient composition, bioactive compounds, food form, and physiological responses [1,13,14]. In contrast, reviews of peanut butter safety have emphasized *Salmonella*, aflatoxins, and their control [7]. Broader reviews of low-moisture foods have concentrated mainly on pathogen persistence and thermal processing [6,16], while food-matrix literature has primarily addressed digestion, nutrient delivery, and health outcomes [11,12,13]. What remains insufficiently synthesized is how the same matrix attributes of peanut butter—particularly low water activity, high lipid content, and semi-solid structure—jointly influence nutritional functionality and hazard behavior. Comparable matrix-based frameworks have been developed for other food categories, most notably dairy foods [17], but have not been applied to peanut butter in a way that integrates nutritional and food safety outcomes.

Accordingly, this review has three objectives: (i) to characterize peanut butter as a nutritionally relevant food matrix; (ii) to explain how matrix properties influence nutrient release, *Salmonella* survival and thermal resistance, and aflatoxin persistence and distribution; and (iii) to translate these relationships into integrated, hazard-specific risk-management priorities across the production chain. The review, therefore, does not treat nutrition and safety as parallel topics but examines them as outcomes shaped by shared physicochemical and structural determinants.

**Figure 1:**
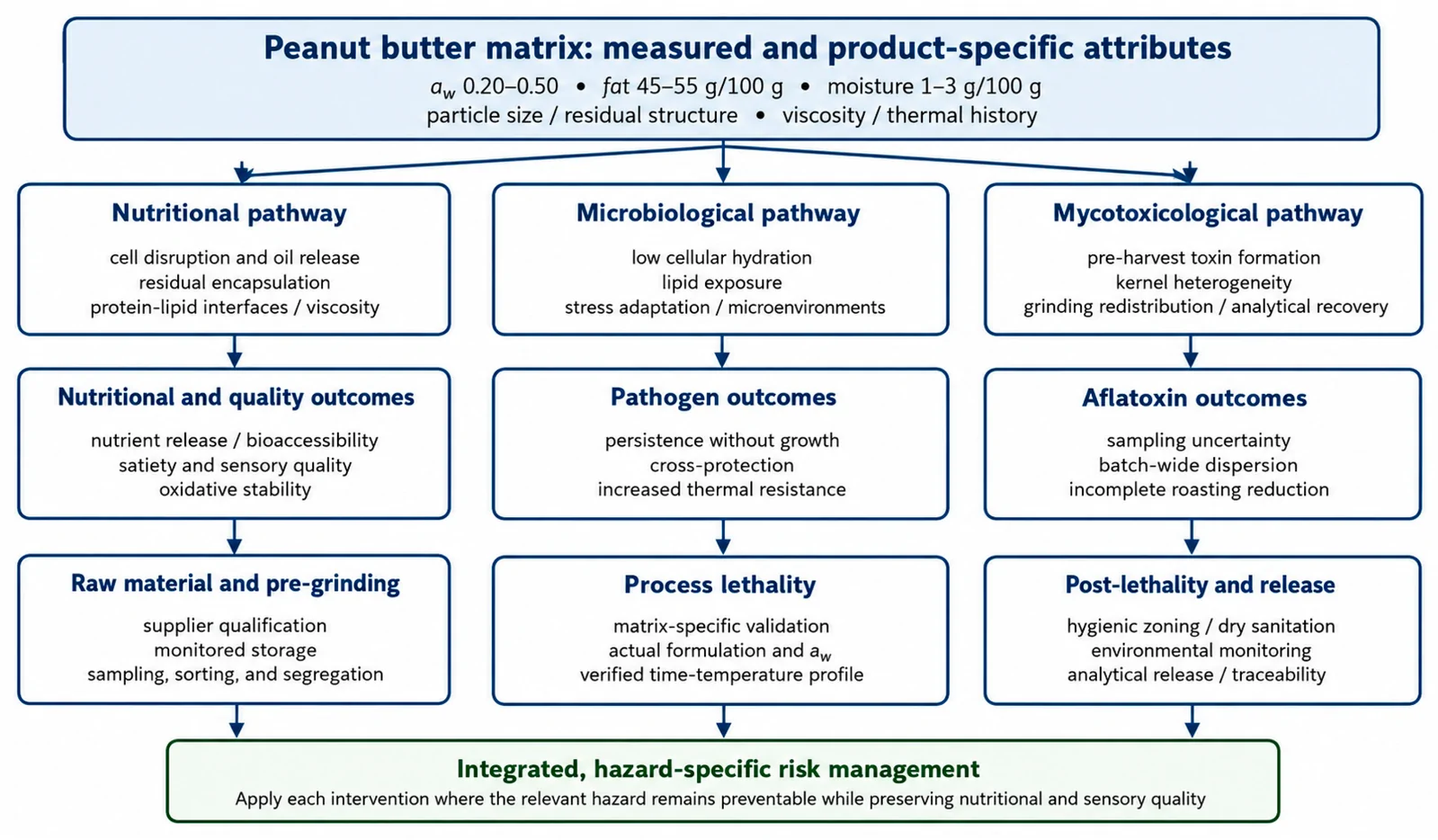
Matrix-based framework linking measurable and product-specific characteristics of peanut butter to nutritional mechanisms, microbiological and mycotoxicological hazard behavior, and stage-specific control points. Matrix attributes influence nutrient release and quality, pathogen persistence and thermal resistance, and aflatoxin distribution and analytical recovery.

Figure 1 translates the food-matrix concept into a sequence of attributes, mechanisms, outcomes, and intervention points. Water activity, lipid content, moisture, particle size, residual cellular structure, viscosity, and thermal history influence nutrient release, cellular hydration and desiccation responses, and the distribution and analytical recovery of aflatoxins. These pathways explain why microbial lethality must be validated in the actual formulation, why aflatoxin controls are most effective before grinding, and why post-lethality hygiene remains essential. In peanut butter, reported D90 °C values were approximately 2–7 min across water activities of 0.20–0.80, with the highest values at the lowest water activity [15].

The contribution of this review should not be judged by the novelty of the diagram itself, but by whether the matrix perspective changes interpretation and control decisions. We use this framework to examine three assumptions that remain common in practice: that shelf stability implies microbiological safety, that nominal composition predicts process lethality, and that roasting can compensate for contaminated raw material. The evidence reviewed below shows that none of these assumptions is reliable across products or hazards.

## 2. Materials and Methods

### 2.1. Literature Search Strategy

A structured literature search was conducted between January and June 2026 using PubMed, Scopus, Web of Science, and Google Scholar. Reference lists of relevant articles and reviews were screened manually, and reports from regulatory agencies and international organizations were included when they provided authoritative evidence on outbreaks, regulatory limits, or risk-management practices.

The search strategy combined three groups of terms: (i) product and matrix terms (“peanut butter”, peanuts, “food matrix”, and “low-moisture foods”); (ii) nutritional terms (“nutritional value”); and (iii) safety and control terms (*Salmonella*, *Escherichia coli*, *Listeria monocytogenes*, and *Clostridium botulinum*, aflatoxins, thermal resistance, microbial persistence, hazard behavior, nonthermal processing, cold plasma, pulsed light, UV-C, UV-LED, risk assessment, and risk management). Boolean operators (“AND” and “OR”) were adapted to the syntax of each database.

Publications from the preceding 10 years were prioritized to capture current evidence. Earlier studies were retained when they were foundational to the understanding of low-moisture food safety, food-matrix effects, microbial survival, thermal resistance, or aflatoxin risk assessment.

**Figure 2:**
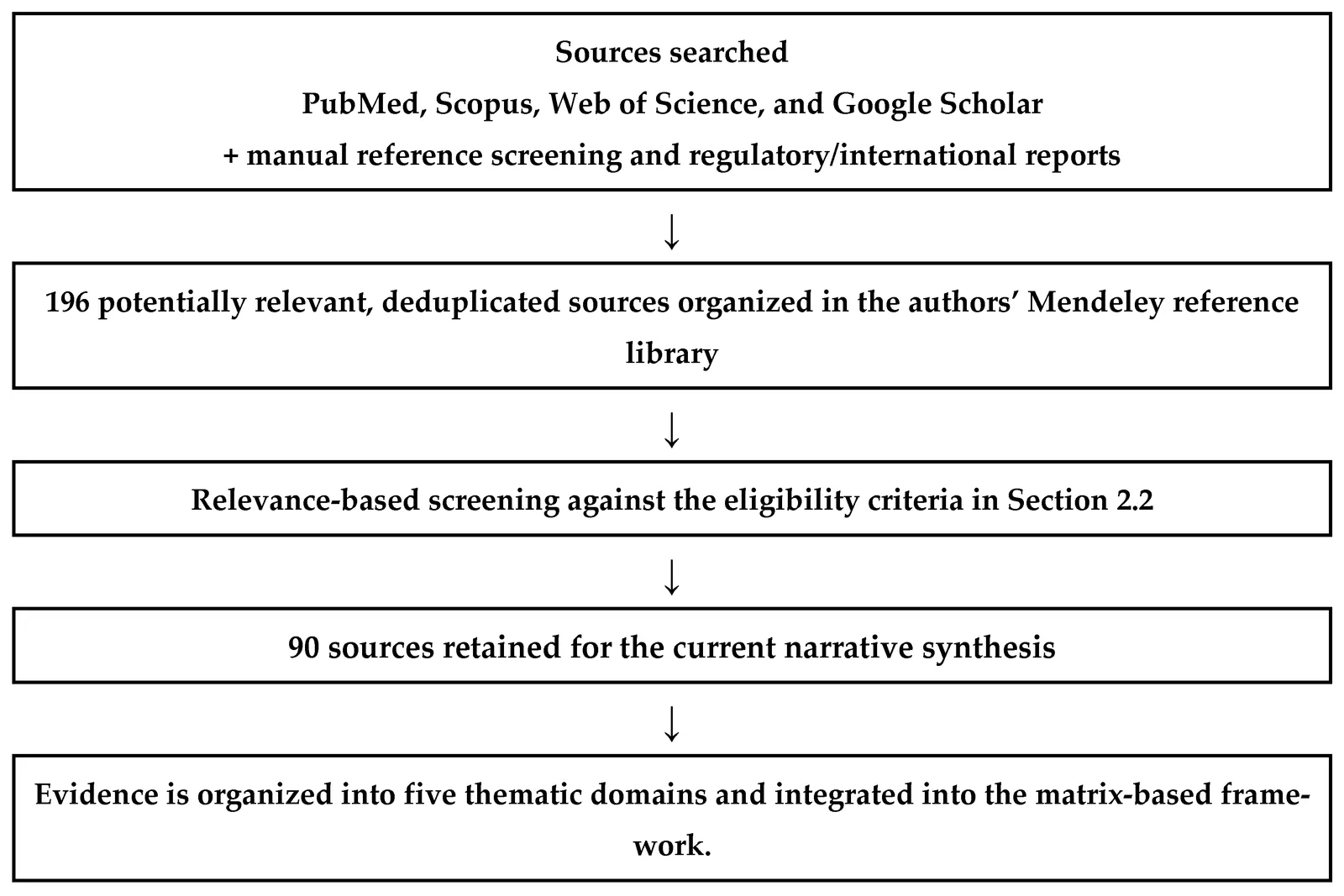
Transparent workflow for literature identification, organization, updating, relevance assessment, and thematic synthesis in this structured narrative review.

During preparation of the original manuscript, 196 potentially relevant, deduplicated sources were organized in the authors’ Mendeley reference library. This number represents the curated candidate library, not the unfiltered count of records returned by the individual databases. Because this was a structured narrative review rather than a systematic review, sources were selected for their conceptual, mechanistic, and practical relevance rather than for quantitative pooling. Following peer review, targeted supplementary searches and reference-list checks were conducted to address the identified mechanistic and comparative gaps, including matrix-mediated digestion, desiccation tolerance, comparisons with other low-moisture foods, secondary microbiological hazards, and emerging control technologies. The current synthesis contains 90 sources represented in the reference list. Figure 2 summarizes the identification, relevance-based selection, and thematic organization of the literature.

### 2.2. Eligibility Criteria and Study Selection

Eligible sources included original research articles, reviews, risk assessments, regulatory documents, guidance reports, and authoritative outbreak reports addressing one or more of the following: peanut butter composition or matrix structure; nutritional functionality; microbial survival, persistence, or inactivation; aflatoxin occurrence and control; or risk-management measures relevant to peanut butter and comparable low-moisture foods.

Sources were excluded when they addressed agronomy, formulation, sensory quality, or allergenicity without a direct link to the food-matrix framework, nutritional functionality, hazard behavior, or risk management. Evidence from other low-moisture foods was retained only when it clarified a mechanism or control principle applicable to peanut butter. Given the narrative design, no formal risk-of-bias tool or quantitative meta-analysis was applied; greater interpretive weight was placed on peer-reviewed mechanistic studies, quantitative evidence, official outbreak investigations, and regulatory guidance. Direct studies conducted in peanut butter were used to support product-specific conclusions, whereas evidence from other low-moisture foods was interpreted as comparative or mechanistic evidence. In vitro digestion studies and emerging-processing experiments were treated as mechanistic or proof-of-principle evidence unless supported by human or industrial validation.

### 2.3. Data Extraction and Narrative Synthesis

The literature was evaluated qualitatively with attention to the food or matrix studied, relevant physicochemical characteristics, nutritional or hazard-related outcomes, processing or storage conditions, and implications for risk management. Evidence was then organized into five thematic areas:

- nutritional composition and dietary relevance of peanut butter;
- food matrix characteristics and their nutritional and technological significance;
- microbiological hazards, with particular emphasis on *Salmonella* survival and thermal resistance, and a targeted assessment of other microbiological hazards;
- mycotoxicological hazards associated with aflatoxin contamination;
- food safety management and risk mitigation strategies across the production chain.

A narrative synthesis was used to integrate evidence across nutrition science, food microbiology, toxicology, food technology, and risk assessment. Studies were compared at the level of mechanisms and practical implications rather than being pooled statistically. Particular emphasis was placed on whether matrix properties altered nutrient release, microbial survival, thermal resistance, contaminant distribution, or the effectiveness of control measures.

The resulting synthesis was used to construct the conceptual framework in Figure 1 and to derive the hazard-specific management priorities discussed in Section 4, Section 5 and Section 6. Because the evidence base includes heterogeneous study designs and regulatory sources, the conclusions are presented as an integrative interpretation rather than as pooled effect estimates.

Evidence was not treated as interchangeable. Outbreak investigations and studies performed directly in peanut butter were used to support product-specific conclusions. In contrast, studies in milk powder, chocolate, flour, spices, oils, or food-contact surfaces were used only to clarify mechanisms or identify testable hypotheses. Likewise, in vitro digestion studies and emerging-processing experiments were interpreted as mechanistic or proof-of-principle evidence unless supported by human or industrial validation.

## 3. Peanut Butter as a Nutritionally Valuable Food Matrix

### 3.1. Nutritional Composition

Peanut butter is a nutrient-dense, lipid-rich food that typically provides 22–30 g/100 g protein, 45–55 g/100 g fat, 4–8 g/100 g dietary fiber, and a range of micronutrients and bioactive compounds [1,18,19,20,21]. Its nutritional significance, therefore, reflects both composition and the physical organization of these components within the product.

Seed-storage proteins dominate peanut proteins. Ara h 1 is a 7S vicilin-type globulin associated with the conarachin fraction, whereas Ara h 3 is an 11S legumin-type globulin associated with arachin; together with Ara h 2, these proteins represent a large proportion of total peanut protein. Nutritionally, peanut protein provides a broad amino acid spectrum, but sulfur amino acids, particularly methionine, are relatively limiting. The same storage proteins include the major peanut allergens. Thermal processing may alter their aggregation and analytical extractability, but it does not reliably eliminate allergenic risk [18,22].

The fiber fraction is heterogeneous and consists predominantly of insoluble cell-wall material, including cellulose, hemicellulose, and lignin, with smaller soluble or pectic fractions. These structures are not merely nutrient entry points: residual cell-wall fragments can retain intracellular lipids and phenolic compounds, thereby modulating their release during digestion. Evidence specific to mucilage and resistant starch in commercial peanut butter remains limited, so these fractions should not be assumed to contribute substantially without product-specific analysis [18,19].

Converting roasted kernels into peanut butter profoundly changes the matrix. Grinding reduces particle size, disrupts cellular structures, releases oil bodies, and modifies viscosity and colloidal stability [23]. Compared with whole peanuts, the butter therefore requires less mastication and presents a larger accessible surface to digestive enzymes. Nevertheless, nutrient release still depends on the extent of residual cellular encapsulation, protein–lipid interfaces, emulsification of the oil phase, and proteolysis of matrix proteins [2,11,13,23].

Matrix effects are also evident during gastric processing. In simulated digestion models, higher-viscosity peanut butter showed slower gastric emptying, protein hydrolysis, and lipid digestion than lower-viscosity formulations [24]. Bioaccessibility should therefore not be inferred from particle size alone. A recent in vitro comparison of whole nuts and their butters further showed that processing altered phenolic concentration and release in a nut- and compound-specific manner rather than producing a uniform increase in bioaccessibility [25].

These studies are mechanistically informative, but two limitations should temper their interpretation. First, in vitro digestion measures bioaccessibility rather than absorbed dose or clinical benefit. Second, much of the health literature focuses on whole peanuts or mixed-nut consumption rather than on commercial peanut butter formulations. Nutritional claims for peanut butter should therefore distinguish product-specific evidence from extrapolation based on composition or related foods [1,20,24,25].

Table 1 summarizes the principal nutritional components of peanut butter and, rather than reproducing composition data alone, links each component to its matrix-dependent nutritional, technological, and safety relevance.

#### 3.1.1. Peanut Butter in Contemporary Dietary Patterns

Contemporary dietary guidance increasingly emphasizes nutrient-dense plant foods, including nuts, legumes, and minimally processed foods. Within this context, peanuts and peanut butter provide unsaturated fatty acids, plant protein, fiber, micronutrients, and bioactive compounds and can contribute to Mediterranean-style and plant-forward dietary patterns [1,3,4,20].

Peanut butter is a practical form of peanut consumption because it is spreadable, energy-dense, and readily consumed by people of all ages. Its ready-to-eat nature is also directly relevant to food safety: consumers typically apply no final lethality step before consumption, so contamination must be prevented and controlled during raw-material sourcing and manufacturing [6,7]. Thus, its dietary relevance strengthens the need for matrix-informed risk management rather than constituting a separate discussion.

#### 3.1.2. Matrix Structure, Digestion, and Safety Implications

Peanut butter can be described as a concentrated, oil-continuous dispersion containing fragmented cell-wall material, protein-rich particles, micronutrients, and bioactive compounds. This organization, rather than composition alone, determines texture, viscosity, phase stability, digestive behavior, and the accessibility of nutrients and hazards [11,12,13,23].

During oral and gastrointestinal processing, matrix breakdown occurs through particle fragmentation, hydration, proteolysis, emulsification, and lipolysis. Butter formation reduces the mastication-dependent fragmentation required for whole kernels, but residual cell walls can still limit intracellular diffusion. Lipid digestion requires enzyme access to the oil–water interface, while protein–lipid interactions and viscosity affect mixing, gastric emptying, and the rate of nutrient release [2,14,23,24].

These nutritional mechanisms have a direct counterpart in food safety. The low water activity of peanut butter prevents the multiplication of most pathogens but does not inactivate them. In combination with the lipid-rich continuous phase, limited water availability can promote long-term persistence and increase *Salmonella’s thermal resistance*, so lethality parameters derived from aqueous foods cannot be transferred directly to peanut butter [6,15,16,26,27,28].

The matrix also influences how hazards are distributed. Aflatoxins are usually formed before peanut butter is manufactured, but grinding can disperse toxins from a small number of contaminated kernels throughout a larger batch. This makes representative sampling, supplier control, sorting, and raw-material verification more important than reliance on finished-product appearance or thermal treatment alone [29,30,31,32].

Allergenicity represents a separate but relevant safety dimension. Major peanut storage proteins remain present in the finished product, and processing-induced changes in aggregation or extractability should not be interpreted as evidence of the elimination of clinical allergenicity. Because the present review focuses on matrix-dependent microbiological and mycotoxicological hazards, allergy is acknowledged here but not reviewed comprehensively [22].

The peanut butter matrix, therefore, has bidirectional consequences: it can enhance accessibility of some nutrients while delaying the release of others, and the same low-moisture, lipid-rich organization can protect hazards or disperse contaminants. This shared mechanistic basis supports the integrated risk-management framework developed in the following sections.

### 3.2. Why Peanut Butter Is a Model System for Matrix-Dependent Hazard Behavior

Peanut butter is a useful model of matrix-dependent hazard behavior not because persistence in low-moisture foods is unique to this product, but because several relevant features converge within one ready-to-eat food: high nutritional density, an oil-continuous semi-solid structure, very low water activity, documented outbreaks, post-roasting handling, and a chemically distinct pre-harvest hazard.

Milk powders, chocolate, flour, spices, and other low-moisture foods share the central paradox that they inhibit bacterial multiplication without necessarily eliminating viable pathogens [6,16]. However, water activity alone does not predict thermal behavior. In a direct isothermal comparison of peanut butter, powdered infant formula, and wheat flour, the predominant order of bacterial thermotolerance was powdered infant formula > peanut butter > wheat flour, despite substantial differences in both water activity and fat content [33]. This demonstrates that proteins, carbohydrates, lipids, physical structure, and the thermal history of the cells act together.

The same principle is evident in other matrices. *Salmonella* survived extended storage in both nonfat dry milk and whole milk powder, whereas rehydration markedly reduced its thermal resistance [34]. In dark, milk, and white chocolate, lower water activity increased survival and D80 °C values, but the magnitude of protection differed among formulations [35]. These findings caution against treating all low-moisture foods as microbiologically equivalent.

Spices provide an additional contrast because their low water activity may coexist with matrix-specific antimicrobial constituents. Transcriptomic analysis of *Salmonella* Typhimurium in milk chocolate, powdered milk, black pepper, and dried pet food showed food-specific regulation during early adaptation, followed by a more convergent low-water-activity stress response [36]. The matrix, therefore, modifies both the intensity and the molecular route of adaptation.

Peanut butter remains distinctive because grinding creates extensive contact with a continuous oil phase, while blending, cooling, filling, and packaging provide opportunities for contamination after roasting [37,38,39]. In parallel, peanuts may carry preformed aflatoxins, whose control depends primarily on agricultural, storage, sorting, sampling, and supplier management measures rather than on the microbial lethality step [8,29,40].

These cross-matrix comparisons clarify mechanisms, but they do not establish a universal ranking of low-moisture foods. Differences in inoculation method, strain, water activity at the treatment temperature, heating system, and recovery medium can alter apparent resistance. Peanut butter is therefore most useful as a test case for product-specific validation, not as a fixed benchmark for all dry foods [16,33,34,35,36].

## 4. Food Safety Challenges Associated with Peanut Butter

Peanut butter challenges several conventional assumptions regarding food safety. Foods with low water activity are generally considered microbiologically stable because these conditions do not support the growth of most pathogenic microorganisms. Consequently, low-moisture products have traditionally been perceived as presenting relatively low microbiological risk. However, numerous outbreaks associated with peanut butter and other low-moisture foods have demonstrated that the absence of microbial growth does not necessarily imply the absence of food safety hazards [7,26].

This apparent contradiction illustrates one of the most important consequences of food matrix characteristics. While the low water activity of peanut butter limits microbial proliferation, the same conditions may facilitate long-term pathogen survival and increase resistance to environmental stresses and processing interventions. Studies have shown that *Salmonella* can persist in peanut butter for extended periods and exhibit enhanced tolerance to thermal treatments under low-moisture conditions [6,15]. Consequently, peanut butter represents a unique example of a food product in which physicochemical properties that contribute to shelf stability simultaneously create challenges for hazard control and risk management [11,12].

In addition to microbiological hazards, peanut butter is associated with chemical risks originating at earlier stages of the production chain. Peanuts are susceptible to contamination by toxigenic fungi, particularly *Aspergillus flavus* and *Aspergillus parasiticus*, which produce aflatoxins, among the most potent naturally occurring foodborne carcinogens [8,41]. Unlike many microbiological hazards that can be substantially reduced through validated processing interventions, aflatoxin contamination frequently originates before harvest or during storage and may persist throughout subsequent manufacturing stages. Effective risk management, therefore, requires an integrated approach encompassing raw material quality, agricultural and storage practices, processing conditions, environmental monitoring, and analytical verification [8,37].

These observations indicate that food safety challenges associated with peanut butter cannot be adequately explained by considering individual hazards in isolation. Instead, they should be understood within the broader context of food matrix characteristics that influence the survival, persistence, resistance, and control of hazards [11]. Therefore, the following sections examine the major biological and mycotoxicological hazards associated with peanut butter and discuss how matrix-dependent behavior influences contemporary food safety management strategies. A brief assessment of additional microbiological hazards is included to distinguish experimentally plausible or population-specific risks from the repeatedly documented outbreak risk posed by *Salmonella*.

### 4.1. Why Salmonella Became the Major Biological Hazard

Among microbiological hazards associated with peanut butter, *Salmonella* is the pathogen of greatest concern. Although peanut butter does not support microbial growth because of its low water activity, numerous studies and outbreak investigations have demonstrated that *Salmonella* can survive for prolonged periods while retaining infectivity. Consequently, it has become the most extensively studied biological hazard in peanut butter and other low-moisture foods.

The public health significance of *Salmonella* is supported by its broad ecological adaptability. The genus comprises more than 2600 serotypes capable of colonizing diverse environmental niches and food matrices [5]. Among those most frequently associated with foodborne illness are *Salmonella enterica* serovars Typhimurium and Enteritidis. Several serovars have also been implicated in outbreaks linked to peanut products, including *S. Typhimurium*, *S. Bredeney*, and *S. Senftenberg* [42,43,44].

Scientific and regulatory attention intensified following a series of major outbreaks associated with peanut-derived products. The most influential event was the 2008–2009 multistate outbreak of *Salmonella typhimurium* linked to products manufactured by the Peanut Corporation of America (PCA), resulting in 714 confirmed illnesses across 46 states, 166 hospitalizations, and 9 deaths [42]. In 2012, a *Salmonella* Bredeney outbreak linked to Sunland Inc. products resulted in 42 confirmed infections across 20 states, approximately 28% of cases requiring hospitalization, and the recall of more than 300 peanut-containing products [43]. More recently, the 2022 Jif outbreak associated with *Salmonella* Senftenberg confirmed that contamination of peanut butter remains a significant food safety challenge despite advances in preventive control systems (Table 2).

The ability of *Salmonella* to persist in peanut butter is closely linked to food matrix characteristics. Experimental studies have demonstrated survival for up to 24 weeks in peanut butter while maintaining infectivity [26]. Unlike many foodborne pathogens, *Salmonella* exhibits pronounced tolerance to desiccation stress. Combined with low water activity and high lipid content, this adaptation creates a protective environment that promotes long-term survival and increases resistance to environmental challenges.

Contamination may occur at multiple stages of production, including during raw peanut handling, in storage facilities, on processing equipment, and in post-roasting environments [38,43]. Consequently, thermal processing alone cannot ensure product safety, as post-process contamination may reintroduce pathogens into finished products [6,42]. Effective control requires an integrated approach combining raw material management, validated roasting procedures, environmental monitoring, hygienic equipment design, and verification programs throughout the production chain [37,43,45,46,47].

Collectively, outbreak investigations and experimental studies have demonstrated that the primary microbiological risk associated with peanut butter arises from pathogen survival and persistence rather than microbial growth [6,26,42]. These findings challenged the traditional assumption that low-moisture foods are inherently microbiologically safe and contributed to the recognition of peanut butter as a valuable model for studying matrix-dependent pathogen behavior and food safety risks in low-moisture food systems [6,7]. Experimental studies also show that *Escherichia coli* O157:H7 can survive in peanut butter and that its persistence and heat resistance vary with formulation, water activity, storage temperature, and prior stress exposure [48].

Table 2 summarizes the major peanut butter-associated *Salmonella* outbreaks, including available evidence on the contamination source, recall scope, regulatory response, and resulting control lessons.

**Table 2:** Major *Salmonella* Outbreaks Associated with Peanut Butter: Source Evidence, Response, and Control Lessons.

Event	Product/Serovar	Impact	Source/Root Cause	Recall/Regulatory Action	Key Control Lesson	Refs
1996 Australia	Peanut butter *Salmonella* Mbandaka	15 cases in South Australia.	Outbreak strain in opened and unopened jars; traceback implicated contaminated roasted peanuts supplied from another Australian state.	Recall volume and number of affected products were not reported in the cited investigation.	Verify suppliers and protect roasted peanuts from recontamination before grinding.	[49]
2006–2007 USA	Peter Pan/Great Value peanut butter *Salmonella* Tennessee	628 cases in 47 states; ~20% hospitalized; no attributed deaths.	Strain recovered from opened and unopened product and two plant environmental samples; the precise route was not established.	All Peter Pan and Great Value products bearing code 2111 were recalled, and production was halted.	A heat step does not prevent environmental or post-process contamination; corrective action requires a root cause investigation.	[50,51]
2008–2009 USA/Canada	PCA peanut butter and paste *Salmonella* Typhimurium	714 cases in 46 U.S. states; 24% hospitalized; nine deaths; one Canadian case.	Contaminated PCA peanut butter and paste entered institutional foods and many downstream products, revealing major preventive-control and ingredient-supply failures.	All products processed since 1 January 2007 were recalled; >2833 downstream products were potentially affected; production stopped.	Supplier verification, traceability, rapid escalation of positive findings, and recall readiness are essential.	[42,52]
2012 USA	Sunland nut butters and peanuts *Salmonella* Bredeney	42 cases in 20 states; 10 hospitalizations; no deaths.	Strain isolated from finished product and the plant environment; FDA found multiple cGMP deficiencies and ineffective internal testing.	Recall expanded to >300 products, including raw and roasted peanuts; FDA suspended the facility registration.	Environmental monitoring must trigger effective investigation, lot disposition, verified corrective actions, and management accountability.	[43]
2022 USA	Jif peanut butter *Salmonella* Senftenberg	21 cases in 17 states; four hospitalizations; no deaths reported.	Strain matched a 2010 plant isolate. FDA identified repeated positives, insufficient corrective actions, and water or unfiltered air entering the post-roast cooling area.	All Lexington products made between 1 October 2021 and 20 May 2022 were recalled; downstream recalls followed; the FDA issued a Warning Letter in 2023.	Post-roast steps require environmental-pathogen hazard analysis, water exclusion, validated corrective actions, and verification beyond finished-product testing.	[44,53,54]

Abbreviations: PCA, Peanut Corporation of America.

Table 2 should be read as a catalog of recurrent failure modes, not as an estimate of current incidence or comparative product risk. Outbreak recognition depends on surveillance, case linkage, traceback, and reporting. The operational signal is nevertheless consistent: post-lethality contamination, persistence in the processing environment, and inadequate response to positive findings can overwhelm an otherwise effective heat step [42,43,44,45,46,47,49,50,51,52,53,54].

### 4.2. Low-Moisture Foods: When Absence of Growth Does Not Mean Absence of Risk

Water activity determines whether microorganisms can multiply, but it is not a direct measure of cell viability. Peanut butter and other low-moisture foods can therefore be shelf-stable while still harboring a small, persistent population of pathogens. For a ready-to-eat product, this distinction is critical because no consumer lethality step is typically applied before ingestion [6,7,16]. Long-term persistence is not exclusive to *Salmonella*; it has also been demonstrated for *Listeria monocytogenes* in nut, seed, and legume butters [55].

During desiccation, water loss reduces cytoplasmic hydration and metabolic activity but can also trigger protective responses. *Salmonella* may accumulate or transport compatible solutes, alter membrane composition, activate the RpoS-controlled general stress response, and induce chaperone and heat-shock systems. These responses stabilize cellular structures and can create cross-protection against subsequent heat, osmotic, acid, and oxidative stresses [36,56,57].

Consequently, persistence is characterized by slow die-off rather than growth [58]. Survival for at least 24 weeks has been demonstrated in peanut butter [26], and viable-cell-selective molecular detection has identified persistent *Salmonella* populations in some peanut products after storage for up to 540 days [59]. These data do not imply multiplication in the finished product; they show that low water activity alone is not a decontamination treatment.

Adaptation is both time- and matrix-dependent. During the initial phase of desiccation, genes associated with osmoprotection, the general stress response, and protein homeostasis may be upregulated; expression patterns can subsequently change as cells enter long-term persistence [57]. Comparative transcriptomics further shows that the early response differs between chocolate, milk powder, black pepper, and other dry foods before a shared core desiccation response becomes more prominent [36].

The physicochemical environment surrounding the cells modifies this response. In peanut butter, the lipid phase, low water activity, limited oxygen, and water mobility, and heterogeneous microenvironments can protect subpopulations differently. Storage temperature, strain or serovar, physiological history, inoculation route, and the recovery method used after stress also influence the measured persistence and should be reported when studies are compared [16,39,56].

Risk management must therefore focus on preventing entry and spread of the pathogen rather than expecting storage to remove it. Hygienic zoning, dry sanitation, environmental monitoring, control of raw materials and rework, and protection of the post-lethality area are essential because a small surviving population may persist throughout the product’s shelf life [37,45,46,47].

### 4.3. Matrix-Dependent Thermal Resistance

Thermal resistance in low-moisture foods is an emergent property of the microorganism, the matrix, and the process. It depends on water activity at the treatment temperature, food composition and structure, strain or serovar, prior desiccation history, inoculation method, heating uniformity, and the recovery procedure used for injured cells [15,16,60]. Consequently, a temperature–time combination developed in an aqueous system cannot be transferred directly to peanut butter.

Limited water availability reduces molecular mobility and changes the pathways through which heat damages proteins, membranes, and other cellular targets. Desiccation-adapted cells can enter heating with compatible solutes, altered membrane properties, and activated stress-response systems, thereby increasing the probability of survival. The result is frequently non-linear inactivation with shoulders or tails rather than a simple log-linear decline [56,57,61].

The lipid phase adds a separate protective contribution. In peanut flour adjusted to the same water activity, exposure to oil during desiccation or heating increased the measured D-values of *Salmonella* Enteritidis, and oil also improved recovery of heat-injured cells during enumeration [39]. Oil protection should therefore not be described only as slower bulk heat transfer; it also reflects the hydration state of cells during heating and the ability of injured survivors to recover.

Cross-protection further connects storage and processing. Desiccation-adapted cells may exhibit enhanced heat resistance, with the magnitude and duration of this response depending on the matrix and storage period [56,57,62,63]. This is consistent with transcriptomic evidence that low-water-activity foods induce both shared stress pathways and matrix-specific gene regulation [36].

Comparative studies show why a universal low-moisture-food lethality model is inappropriate. Under matched experimental methods, thermotolerance was generally greater in powdered infant formula than in peanut butter and wheat flour [33]. In chocolate, reducing water activity from 0.50 to 0.25 increased D80 °C values, but the effect differed among dark, milk, and white formulations [35]. In milk powder, hydration substantially reduced thermal resistance [34]. Thus, neither fat content nor room-temperature water activity alone adequately predicts process lethality.

Validation should therefore reproduce the actual product formulation, water activity over the relevant temperature range, the expected contamination route, the heating profile, the equipment configuration, and a suitably resistant target or surrogate [60,64]. Roasting can provide substantial reductions when it is properly designed and verified, but no generic temperature range can replace product- and process-specific validation [60,61,65,66]. Post-roasting segregation remains equally important because a validated lethality step cannot compensate for recontamination. Aflatoxins require a different strategy, as discussed below, because their prevention begins before manufacture, and thermal processing provides only partial reduction.

### 4.4. Other Microbiological Hazards: Secondary and Population-Specific Risks

Although *Salmonella* remains the dominant outbreak-associated bacterial hazard, *Escherichia coli* O157:H7 can also persist in peanut butter under experimental conditions. In a 30-day study, survival and subsequent heat resistance varied with product formulation, water activity, storage temperature, and prior stress exposure [48]. No peanut butter outbreak caused by *E. coli* O157:H7 was identified in the evidence included in this review; its relevance is therefore primarily as a secondary contamination hazard and as evidence that matrix protection is not specific to *Salmonella*.

Among *Clostridium* species, *Clostridium botulinum* warrants a distinct, population-specific interpretation. In a confirmed United Kingdom case in 2024, type A *C. botulinum* was recovered from both the feces of a 6-month-old infant and the peanut butter consumed, and whole-genome sequencing identified matching strains [67]. The event represented infant botulism, in which ingested spores can germinate in the immature intestinal tract; it should not be interpreted as evidence of toxin formation in the peanut butter or as a comparable risk for the general adult population.

*Listeria monocytogenes* can likewise persist for prolonged periods in nut, seed, and legume butters [55], although no peanut butter-associated outbreak was identified in the reviewed evidence. These findings support broad controls for raw materials, post-process exposure, dry sanitation, and environmental monitoring. Still, they do not displace *Salmonella* as the principal microbiological hazard because repeated outbreaks, environmental persistence, and matrix-enhanced heat resistance are documented most consistently for this pathogen.

## 5. Mycotoxicological Hazards and Pre-Processing Risk Factors

Unlike microbiological hazards, which may be introduced, survive, or spread during processing, mycotoxicological hazards often originate before manufacturing begins. In peanut butter production, aflatoxins represent the most important example of this fundamentally different risk pathway.

### 5.1. Aflatoxins: A Hazard Originating Before Processing

Aflatoxins follow a fundamentally different risk pathway from *Salmonella*. They are usually formed before peanut butter manufacture, during crop development, harvest, drying, transport, or storage. Once present, the toxins may persist even when the producing fungus is no longer viable, so absence of visible mold does not demonstrate product safety [8,9,68,69].

The principal food-relevant aflatoxins are AFB_1_, AFB_2_, AFG_1_, and AFG_2_, produced mainly by *Aspergillus flavus* and *A. parasiticus*. AFB_1_ is generally the most toxic and is the component specifically limited alongside the sum of the four aflatoxins in European legislation [40,70,71].

Chronic AFB_1_ exposure is associated with genotoxicity, immunotoxicity, hepatotoxicity, and hepatocellular carcinoma, and AFB_1_ is classified as carcinogenic to humans. The risk is cumulative, which supports prevention and exposure reduction throughout the supply chain rather than reliance on a single end-stage intervention [41,70,71,72,73].

Aflatoxin contamination is also highly heterogeneous. A small number of kernels may carry a large proportion of the lot burden, while apparently normal kernels may still be contaminated. Grinding then redistributes residual toxin through a larger mass of peanut butter. Representative sampling and validated analysis are therefore essential; visual inspection alone is insufficient [29,30,31,32,68,74].

Regulatory limits depend on the intended stage of use. Under the current European Union framework, peanuts intended for sorting or other physical treatment may contain up to 8 μg/kg AFB_1_ and 15 μg/kg total aflatoxins. In contrast, peanuts and peanut products placed on the market for the final consumer are limited to 2 μg/kg AFB_1_ and 4 μg/kg total aflatoxins. “Total aflatoxins” denotes the sum of AFB_1_, AFB_2_, AFG_1_, and AFG_2_. In the United States, the FDA applies an action level of 20 μg/kg (20 ppb) for total aflatoxins in human foods, including peanuts and peanut products [40,75].

These limits do not imply that lots close to the legal threshold are technically equivalent to low-contamination lots. Because the distribution is heterogeneous and sampling error can be substantial, risk management should combine supplier qualification, representative lot sampling, validated analytical methods, removal of visibly or physically defective kernels, and process controls that prevent further fungal activity [30,31,32,68,76].

The central distinction is therefore temporal and mechanistic: *Salmonella* is controlled mainly through exclusion, validated lethality, sanitation, and protection of the post-process environment, whereas aflatoxin control begins in the field and continues through drying, storage, sorting, sampling, and analytical release of raw materials [7,8,9,30,68,76].

### 5.2. Why Thermal Processing Is Not a Sufficient Control Strategy

Aflatoxins are comparatively heat-stable small molecules rather than living targets. Conventional roasting can promote chemical degradation, but it does not provide the predictable log-reduction concept used for microbial lethality. Residual toxin may remain after severe heating, and apparent concentration changes can also be influenced by matrix composition and analytical recovery [71,77].

Quantitative peanut-roasting data illustrate this limitation. In one controlled study, treatments at 160, 180, and 200 °C for up to 25 min produced maximum aflatoxin reductions of 61.6%, 83.6%, and 89.7%, respectively. At 180 °C for 20 min, reductions ranged from 55% to 81% across different initial contamination levels. Even the most intensively tested conditions, therefore, left a measurable residual fraction [77].

Reported percentage reductions should not be compared as if they were intrinsic properties of roasting. Initial contamination, moisture loss, heating uniformity, matrix composition, and analytical recovery can all change the apparent reduction. For risk management, the relevant question is not the maximum percentage observed in a laboratory experiment, but whether a compliant incoming lot remains under control within a validated commercial process. A highly contaminated lot cannot be made acceptable by relying on an average percentage reduction [30,31,32,77].

Physical removal before grinding is more effective than attempting to treat the toxin after it has been dispersed throughout the butter. Size and density separation, gravity systems, shelling and blanching inspection, manual or electronic color sorting, and optical or hyperspectral screening can remove kernels associated with visible, structural, or spectral abnormalities. However, contaminated kernels are not always visibly defective, and sorter performance is lot- and technology-dependent [30,31,32,74].

No single sorting or testing method should be presented as universally superior. Rapid strip or lateral-flow tests can support lot screening, while chromatographic or validated immunochemical methods provide quantitative verification. Because analytical accuracy cannot compensate for an unrepresentative sample, the control sequence should be risk-based: supplier approval and lot segregation, representative sampling, physical or optical sorting where justified, rapid screening, and confirmatory release testing [30,74,76].

Roasting remains valuable as one component of a cumulative control system, but its primary validated food-safety function is microbial lethality. For aflatoxins, the decisive controls are prevention of formation, rejection or segregation of unsuitable lots, removal of high-risk kernels, and analytical verification before contaminated material is irreversibly mixed by grinding [29,30,68,76,77].

### 5.3. Agricultural and Storage Determinants of Aflatoxin Formation

Prevention must begin before manufacture. Field stress, harvest timing, kernel damage, drying rate, storage temperature, relative humidity, kernel moisture, insects, and storage duration interact to determine whether fungal colonization progresses to toxin production [8,68,78].

Hot, dry conditions during crop development can weaken plant defenses and increase susceptibility to *A. flavus*, while subsequent humidity during curing or storage permits fungal activity. Field management, therefore, includes timely harvest, reduction in drought and insect stress where feasible, exclusion of damaged pods, and use of validated atoxigenic biocontrol strains in regions where such programs are authorized [9,79,80,81].

Insect infestation and mechanical damage increase access to the kernel and create local conditions favorable to fungal invasion. Post-harvest studies in U.S. peanut storage facilities have linked higher temperatures and kernel moisture with greater insect activity and aflatoxin contamination, demonstrating that storage design and monitoring affect both quality and safety [81,82].

Experimental work on stored peanuts found aflatoxin formation under relative humidities of 83–86%, depending on kernel condition and temperature, and generally associated the formation with kernel moisture contents of approximately 10% or higher [83]. These values are not universal operating limits, but they show why delayed drying and local moisture migration are hazardous. Modern storage programs should therefore continuously monitor temperature and relative humidity, verify kernel moisture, identify hot spots, maintain aeration where appropriate, and trigger corrective action when trends indicate condensation or moisture ingress [68,78,82,83].

Climate-related risk is no longer confined to traditionally tropical production zones. A European modeling study projected that a +2 °C scenario could substantially increase AFB_1_ risk in maize, illustrating how aflatoxigenic hazards may emerge in temperate regions [9]. Although this evidence is crop-specific and should not be transferred quantitatively to peanuts, it supports climate-responsive sourcing, surveillance, and storage plans rather than fixed historical assumptions [9,84].

Accordingly, preventive programs should link field and supplier information with incoming-lot testing and storage records. Lots from drought-affected regions, delayed harvests, damaged kernels, or adverse storage histories warrant intensified sampling, segregation, and verification rather than routine acceptance [68,76,78,80].

### 5.4. Integrated Prevention and Risk Management

Effective control requires a coordinated hierarchy rather than a single critical step. The hierarchy begins with approved suppliers and agricultural prevention, continues through drying and monitored storage, and then applies lot segregation, representative sampling, sorting, processing controls, analytical release, traceability, and recall readiness [7,8,9,30,68,76].

For *Salmonella*, the manufacturing priorities are a validated roasting process, hygienic zoning, dry sanitation, environmental monitoring, hygienic equipment design, and physical separation of raw and post-lethality areas. Continuous and batch roasting can both be effective when the actual time–temperature distribution and worst-case product conditions are validated; neither configuration is inherently reliable without process control and verification [60,65,66].

For aflatoxins, the most effective combination depends on lot risk, production scale, throughput, and technical capacity. High-throughput plants may justify in-line optical sorting and automated sensor systems, whereas smaller operators may rely more heavily on qualified suppliers, outsourced analytical testing, and strict rejection criteria. In all cases, visual sorting or rapid tests must remain subordinate to representative sampling and validated quantitative verification [30,31,32,68,74,76].

Legal maximum or action levels are release criteria, not process targets. Food business operators should maintain contamination at the lowest reasonably achievable level through preventive controls and trend analysis. Plant sanitation does not destroy preformed aflatoxins, but clean, dry raw-material areas and the control of leaks, condensation, pests, and rework reduce conditions that could permit further fungal activity, or cross-lot spread [40,75,76].

The peanut butter matrix determines when interventions remain effective. Sorting and representative sampling are most informative before grinding, while the continuous lipid phase and low water activity must be reproduced during microbial process validation. Once kernels are comminuted and mixed, residual aflatoxin becomes more widely distributed and cannot be reliably managed by appearance or roasting [11,29,30].

An integrated program must therefore apply different controls at the stages where each hazard is still preventable: field and storage controls for aflatoxin formation, lot sampling and sorting before grinding, validated lethality and environmental control for microbial hazards, and analytical verification before product release. This stage-specific approach is more defensible than expecting one processing intervention to control all hazards [7,8,16,76].

## 6. Balancing Nutritional Value and Food Safety

Having examined nutritional composition and hazard behavior separately, this discussion now brings the two perspectives together within the food matrix framework introduced at the outset of this review. Table 3 summarizes the principal differences between *Salmonella* and aflatoxins as food safety hazards, illustrating why a single generic control strategy is insufficient and why risk management must instead account for the shared matrix properties driving both nutritional functionality and hazard persistence.

### 6.1. Nutritional Benefits as the Basis for Risk–Benefit Evaluation

Peanut butter provides a concentrated combination of plant protein, predominantly unsaturated lipids, dietary fiber, micronutrients, phytosterols, and phenolic compounds [1,2,18,19,20]. Its nutritional value depends not only on composition but also on formulation and matrix structure, which influence nutrient release, satiety, and bioaccessibility.

The nutritional case is credible but should not be overstated. Much of the clinical evidence concerns peanuts, nuts, or dietary patterns rather than commercial peanut butter itself [1,20]. Product formulation, portion size, added sugars, sodium, and stabilizers can modify the practical nutritional profile, so benefits demonstrated for whole nuts should not be transferred uncritically to every peanut butter product.

Processing also changes nutritional functionality. Compared with intact kernels, peanut butter requires less mastication. It exposes a larger surface to digestive processes, yet residual cell structures, viscosity, and protein–lipid interfaces can still limit or delay the release of specific components [14,23,24,25].

A risk–benefit perspective, therefore, does not justify tolerating contamination. Rather, it requires safety interventions that achieve the necessary hazard control while avoiding unnecessary losses of nutritional quality, oxidative stability, texture, and consumer acceptability.

Our interpretation is therefore conditional: peanut butter can contribute useful nutrients, but nutritional value does not offset a preventable safety failure. Product and process design should preserve desirable nutritional and sensory properties only within a control system that first meets microbiological and chemical safety requirements.

### 6.2. Food Safety Risks Cannot Be Ignored

The principal microbiological concern is *Salmonella*, because the organism can persist without growth and may acquire substantial heat resistance in low-water-activity, lipid-rich matrices [6,15,26,27,28]. Process validation must therefore reproduce the actual formulation, water activity, heating profile, and post-lethality contamination routes.

Aflatoxins require a different control logic. They generally originate before manufacture, are heterogeneously distributed among kernels, and are not reliably eliminated by roasting [8,9,29,77,85,86]. The evidence also supports a hierarchy of concern: recurrent outbreaks and direct peanut-butter challenge studies justify prioritizing *Salmonella;* experimental survival of *Escherichia coli* and *Listeria monocytogenes* establishes biological plausibility; and the botulism report defines a narrow infant-specific scenario. Treating these evidence levels as equivalent would overstate secondary hazards and understate the pathogen with the strongest product-specific record [42,43,44,45,46,47,48,49,50,51,52,53,54,55,67].

### 6.3. Food Matrix as the Link Between Benefits and Risks

The food matrix is the mechanistic link between nutritional function and hazard behavior. It governs nutrient accessibility, the local hydration state of microorganisms, contact with the lipid phase, contaminant redistribution during grinding, and the product’s response to processing.

Low water activity contributes to shelf stability but does not sterilize the product; the lipid-rich continuous phase can further protect desiccated cells during heating [15,26,27,28]. Conversely, grinding can improve access to some nutrients while dispersing aflatoxin from a small number of contaminated kernels through a much larger batch [25,29,30,31,32].

These mechanisms determine when controls are most effective: sorting and representative sampling must precede grinding, microbial lethality must be validated in the real matrix, and post-roasting zones must be protected from environmental recontamination.

In practical terms, matrix characterization is useful only when it changes a validation protocol, sampling plan, or release decision. Reporting fat content and water activity without linking them to process performance adds description, but not risk-management value.

### 6.4. Toward a Risk–Benefit Perspective

A formal quantitative risk–benefit analysis is not currently possible because nutritional and safety outcomes are measured on incompatible scales and are rarely studied in the same products or populations. We therefore use risk–benefit reasoning qualitatively: safety thresholds remain non-negotiable, while alternative controls can be compared for avoidable effects on oxidation, texture, nutrient accessibility, and consumer acceptance.

This requires stage-specific priorities: agricultural and storage prevention for aflatoxins; supplier verification, sampling, and sorting before grinding; validated roasting and hygienic segregation for microbial hazards; and analytical or environmental verification before product release.

Interventions should be evaluated against both safety and quality endpoints. For example, increasing process intensity may improve microbial lethality but can also alter lipid oxidation, sensory quality, protein structure, and bioactive compounds; these effects must be measured rather than assumed.

This interpretation is deliberately conservative. The framework is intended to improve the choice and placement of controls, not to trade a measurable food-safety hazard against a general nutritional benefit.

### 6.5. Lessons Beyond Peanut Butter

The core lesson is transferable to other low-moisture foods: absence of growth cannot be equated with absence of viable pathogens, and the effectiveness of a control depends on the local matrix rather than on nominal water activity or fat content alone [6,16,33,34,35,36].

However, transferability should not be assumed. Milk powders, chocolate, flour, spices, and nut-based products differ in composition, microstructure, routes of contamination, and processing histories, so each requires product-specific evidence.

Peanut butter is valuable as a model because it combines a ready-to-eat low-moisture matrix with documented post-lethality bacterial contamination and a chemically stable pre-harvest toxin.

The framework can guide hypothesis generation and the design of validation studies in other products, but it should not be used as a substitute for product-specific data. Its transferability is an empirical question.

### 6.6. Future Perspectives

The immediate research priority is not another broad screening study, but validation under industrially realistic conditions. Predictive models should incorporate formulation, water activity at the treatment temperature, local microstructure, prior stress history, and the actual heating profile, and they should be challenged across production lots, strains, equipment configurations, and recovery methods. A model that performs only in a single laboratory matrix has limited value for process authority decisions.

Nonthermal technologies remain proof-of-principle rather than established peanut-butter controls. Cold plasma has inactivated *Aspergillus flavus* and reduced spiked AFB_1_ on raw peanuts [87]; UV-LED has degraded AFB_1_ in peanut oil [88]; UV-C has reduced dry-adhered *Salmonella* on food-contact surfaces [89]; and pulsed light has impaired *Salmonella* culturability in model surface systems [90]. None of these results, taken alone, demonstrate uniform treatment of a flowing or packaged peanut butter matrix.

The next useful experiments should measure safety and quality endpoints in the same design: microbial or toxin reduction, treatment uniformity, penetration or shadowing, throughput, degradation-product toxicology, lipid oxidation, sensory change, and nutrient retention. Until such evidence becomes available, nonthermal methods should be treated as supplementary measures, not as alternatives to supplier control, representative sampling, validated roasting, hygienic zoning, and the prevention of aflatoxin formation. Climate-responsive sourcing and storage surveillance remain parallel priorities [9,84].

### 6.7. Strengths and Limitations

The main strength of this review is the explicit comparison of evidence that is usually kept in separate disciplinary bodies of literature. Outbreak investigations, direct peanut butter challenge studies, aflatoxin control evidence, digestion studies, and regulatory requirements were not summarized as equivalent facts; they were used for different purposes within a single matrix-based interpretation.

The limitations are substantial and define how the conclusions should be used. This is a structured narrative rather than a systematic review, so the source-selection process cannot be used to estimate completeness, publication bias, or pooled effect sizes. No included study simultaneously measured nutritional effects, pathogen behavior, and aflatoxin control in the same peanut butter formulation; the central framework is therefore an inferential synthesis of separate evidence streams. Much of the outbreak evidence comes from the United States, and the regulatory comparison emphasizes EU and U.S. requirements. Reported D-values and aflatoxin reductions vary with strain, matrix, water activity, analytical recovery, and process conditions and should not be treated as universal constants. Evidence for nonthermal technologies remains largely at the laboratory scale, and extension to other low-moisture foods requires direct validation.

## 7. Conclusions

Our critical synthesis supports three conclusions. First, shelf stability is not a microbiological control: *Salmonella* can persist without growth, and matrix-dependent heat resistance makes product-specific validation essential. Second, aflatoxin risk is primarily a raw-material and pre-grinding problem; roasting may reduce contamination, but cannot reliably correct a non-compliant lot. Third, the food-matrix concept has practical value only when it changes where samples are taken, how lethality is validated, or when a lot is rejected or released.

The defensible control strategy is therefore stage- and hazard-specific: prevention and monitored storage for aflatoxins; representative sampling and sorting before grinding; validated lethality in the actual formulation; protection of post-roasting areas; and verification before release. Nutritional and sensory quality should be measured alongside safety during process development, but neither should justify weaker controls. Emerging nonthermal technologies may eventually add useful hurdles; at present, their role in finished peanut butter remains to be demonstrated.

## Figures and Tables

**Table 1:** Major Components of Peanut Butter and Their Matrix-Dependent Nutritional and Safety Relevance.

Component	Typical Content	Nutritional Role	Matrix-Dependent Interpretation	Key References
Protein	22–30 g/100 g	Plant protein contributes to satiety and protein intake	Dominated by storage proteins, including Ara h 1 and Ara h 3. Protein particles and aggregates contribute to water binding, rheology, digestibility, and allergenic risk.	[18,22]
Total fat	45–55 g/100 g	Energy source and carrier of fat-soluble nutrients	Forms the continuous lipid phase. Grinding releases oil bodies and alters viscosity; the lipid phase also influences oxidation and can protect microorganisms during heating.	[18,21,23]
MUFA	22–28 g/100 g	Major unsaturated lipid fraction associated with favorable cardiometabolic profiles	Contributes to lipid-phase fluidity, nutrient solubilization, sensory properties, and oxidative stability.	[1,18,20]
PUFA	12–18 g/100 g	Source of essential fatty acids	Supports nutritional quality but increases susceptibility to lipid oxidation and related shelf-life changes.	[1,18,19]
Dietary fiber	4–8 g/100 g	Supports satiety and gastrointestinal function	Cell-wall material and residual cellular fragments influence particle integrity and the release of intracellular lipids and phenolics.	[18,19,25]
Available carbohydrates	15–22 g/100 g	Additional energy source	Contribute to the dispersed solid phase, although their structural role is smaller than that of lipids, proteins, and cell-wall material.	[18,19]
Moisture content	1–3 g/100 g	Limited direct nutritional contribution	Affects consistency, physical stability, oxidation, and the water activity of the finished product.	[7,21]
Water activity (*a*_w_)	0.20–0.50	Not a nutrient; describes available water	Restricts microbial growth but may favor long-term persistence and increased thermal resistance of pathogens.	[16,26,27]

Abbreviations: *a*_w_, water activity; MUFA, monounsaturated fatty acid; PUFA, polyunsaturated fatty acid.

**Table 3:** Comparison of the Two Major Food Safety Hazards Associated with Peanut Butter and Their Implications for Risk Management.

Characteristic	*Salmonella*	Aflatoxins	Food Safety Implication	Key References
Hazard type	Biological	Chemical	Different hazard categories require distinct control and monitoring strategies	[16,71]
Principal causative agent	*Salmonella enterica* serovars	Aflatoxins (AFB_1_, AFB_2_, AFG_1_, AFG_2_) produced mainly by *Aspergillus flavus* and *A. parasiticus*	Control measures must target either microorganisms or their toxic metabolites	[16,71]
Primary source of contamination	Raw materials and processing environment	Field contamination and post-harvest storage	Preventive controls must focus on different stages of the supply chain	[16,71]
Main stage of occurrence	Pre- and post-processing	Primarily pre-harvest and storage	Hazard prevention cannot rely on a single control point	[71,76]
Ability to multiply in peanut butter	No	Not applicable	Absence of growth does not imply absence of risk	[16]
Persistence in peanut butter	Long-term survival (months to years)	Long-term chemical stability	Hazards may remain despite prolonged storage	[26,71]
Influence of food matrix	Strong; low *a*_w_ and high fat enhance survival and thermal resistance	Highly heterogeneous among kernels before grinding; grinding redistributes residual toxin, and matrix composition affects analytical recovery	Sampling, sorting, and matrix-appropriate analytical recovery are integral to risk assessment	[11,30,71,74]
Public health outcome	Acute gastroenteritis, hospitalization, invasive infection	Chronic toxicity, hepatocellular carcinoma, immunotoxicity	Risk management must address both acute and chronic health effects	[5,41,70,72,73]
Effectiveness of roasting	Significant reduction when properly validated	Variable and incomplete reduction; measurable toxin may remain even after intensive roasting	Roasting is a supporting hurdle, not a corrective treatment for non-compliant lots.	[26,77]
Primary control strategy	Process validation, environmental monitoring, and hygienic design	Prevention, supplier verification, GAP, and storage management	Hazard-specific preventive controls are required	[43,76]
Most critical intervention point	Manufacturing environment	Agricultural production and storage	Different hazards require different intervention priorities	[43,71]
Verification methods	Microbiological testing, environmental monitoring, WGS	HPLC, LC-MS/MS, ELISA	Verification methods must reflect hazard characteristics	[44,76]
Regulatory focus	Prevention of pathogen contamination	EU maximum levels for AFB_1_ and total aflatoxins; U.S. action level for total aflatoxins	Compliance criteria differ by jurisdiction and intended stage of use	[40,75]
Key food safety lesson	Survival can occur without growth.	The absence of visible fungal contamination does not guarantee the absence of toxins.	Effective risk management requires preventive rather than reactive approaches.	[16,71]

Abbreviations: *a*_w_, water activity; AFB_1_/AFB_2_/AFG_1_/AFG_2_, aflatoxins B_1_, B_2_, G_1_, and G_2_; GAP, good agricultural practices; HPLC, high-performance liquid chromatography; LC-MS/MS, liquid chromatography–tandem mass spectrometry; ELISA, enzyme-linked immunosorbent assay; WGS, whole-genome sequencing.

## Data Availability

No new data were created or analyzed in this study. Data sharing is not applicable to this article.
